# Capsule endoscopy findings for the diagnosis of Crohn’s disease: a nationwide case–control study

**DOI:** 10.1007/s00535-018-1507-6

**Published:** 2018-09-15

**Authors:** Motohiro Esaki, Takayuki Matsumoto, Naoki Ohmiya, Ema Washio, Toshifumi Morishita, Kei Sakamoto, Hiroo Abe, Shojiro Yamamoto, Tetsu Kinjo, Kazutomo Togashi, Kenji Watanabe, Fumihito Hirai, Masanao Nakamura, Sadaharu Nouda, Shinya Ashizuka, Teppei Omori, Shuji Kochi, Shunichi Yanai, Yuta Fuyuno, Atsushi Hirano, Junji Umeno, Takanari Kitazono, Fukunori Kinjo, Mamoru Watanabe, Toshiyuki Matsui, Yasuo Suzuki

**Affiliations:** 1 Department of Medicine and Clinical Science, Graduate School of Medical Sciences, Kyushu University, Maidashi 3-1-1, Higashi-ku, Fukuoka, 812-8582 Japan; 2 Division of Gastroenterology, Department of Internal Medicine, School of Medicine, Iwate Medical University, Morioka, Japan; 3 Department of Gastroenterology, Fujita Health University School of Medicine, Toyoake, Japan; 4 Department of Gastroenterology and Hematology, Faculty of Medicine, University of Miyazaki, Miyazaki, Japan; 5 Department of Endoscopy, University of Ryukyus Hospital, Nishihara, Okinawa Japan; 6 Department of Coloproctology, Aizu Medical Center, Fukushima Medical University, Aizuwakamatsu-City, Japan; 7 Department of Intestinal Inflammation Research, Hyogo College of Medicine, Nishinomiya, Hyogo Japan; 8 Inflammatory Bowel Disease Center, Fukuoka University Chikushi Hospital, Chikushino, Japan; 9 Department of Gastroenterology and Hepatology, Nagoya University Graduate School of Medicine, Nagoya, Japan; 10 Second Department of Internal Medicine, Osaka Medical College, Takatsuki, Japan; 11 Department of Circulatory and Body Fluid Regulation, Faculty of Medicine, University of Miyazaki, Miyazaki, Japan; 12 Institute of Gastroenterology, Tokyo Women’s Medical University, Tokyo, Japan; 13 Division of Gastroenterology, Matsuyama Red Cross Hospital, Matsuyama, Japan; 14 Center for Gastroenterology, Urasoe General Hospital, Urasoe, Japan; 15 Department of Gastroenterology, School of Medicine, Tokyo Medical and Dental University, Tokyo, Japan; 16 Department of Internal Medicine, Toho University, Sakura Medical Centre, Sakura, Chiba Japan

**Keywords:** Capsule endoscopy, Crohn’s disease, Diagnostic accuracy

## Abstract

**Background:**

Capsule endoscopy can be used to identify the early stage of small bowel Crohn’s disease (CD). We evaluated significant small bowel capsule endoscopy (SBCE) findings that can lead to early diagnosis of CD.

**Methods:**

We retrospectively accumulated clinical and SBCE data of 108 patients (63 with and 45 without CD). Types of small bowel mucosal injuries, including erosion, ulceration, and cobblestone appearance, and the alignment of diminutive lesions were compared between patients with and without CD. Inter- and intra-observer agreement in the determination of lesions was assessed in 25 pairs of SBCE from the two groups.

**Results:**

Under SBCE, cobblestone appearance (33% vs. 2%, *p* < 0.0001), longitudinal ulcers (78% vs. 20%, *p* < 0.0001), and irregular ulcers (84% vs. 60%, *p* < 0.01) were more frequently found in patients with CD. Linear erosion (90% vs. 38%, *p* < 0.0001) and irregular erosion (89% vs. 64%, *p* < 0.005) were also more frequent in patients with CD. Furthermore, circumferential (75% vs. 9%, *p* < 0.0001) and longitudinal (56% vs. 7%, *p* < 0.0001) alignment of diminutive lesions, mainly observed in the 1st tertile of the small bowel, was more frequent in patients with CD. Good intra-observer agreement was found for ulcers, cobblestone appearance, and lesion alignment. However, inter-observer agreement of SBCE findings differed among observers.

**Conclusions:**

Circumferential or longitudinal alignment of diminutive lesions, especially in the upper small bowel, may be a diagnostic clue for CD under SBCE, while inter-observer variations should be cautiously considered when using SBCE.

**Electronic supplementary material:**

The online version of this article (10.1007/s00535-018-1507-6) contains supplementary material, which is available to authorized users.

## Introduction

As evidence has accumulated regarding the use of small bowel capsule endoscopy (SBCE), this procedure has been considered useful for the diagnosis of small bowel pathologies [1–4]. SBCE is invaluable for identification of small bowel lesions, mucosal healing, and postoperative recurrence in patients with suspected or established diagnosis of Crohn’s disease (CD) [5–10].

While SBCE is not a standard technique for the diagnosis of CD, several reports have suggested favorable diagnostic yields of the procedure in patients with suspected CD [11–15]. A meta-analysis also suggested the superiority of SBCE for the diagnosis of CD in comparison with small bowel radiography, push enteroscopy, and magnetic resonance enterography [16]. However, the criteria for SBCE-based diagnosis of CD vary widely among previous clinical trials.

Mow et al. [17] suggested that > 3 ulcerations under SBCE and a negative history for the use of nonsteroidal anti-inflammatory drugs (NSAIDs) are diagnostic for CD in patients suspected to have this disease. However, considering the characteristic mucosal lesions in patients with CD, such as longitudinal ulcers and cobblestone appearance [18–21], the types and predominant location of mucosal lesions need to be seriously considered when establishing SBCE-based diagnosis of CD. Furthermore, diminutive mucosal lesions such as aphthous lesions or erosions have been considered the precursor lesions of CD [18, 19, 22]. In particular, the lesions aligned longitudinally develop into longitudinal ulcers with the progress of the disease [22]. However, no other characteristic findings are determined to date that help discriminate the early stage of CD from other miscellaneous diseases. In this sense, SBCE can be useful because it is superior to other modalities for the depiction of diminutive mucosal lesions of the small bowel.

In the present study, we aimed to identify the SBCE findings that lead to the diagnosis of the early stage of CD. Furthermore, inter-observer and intra-observer variations in SBCE findings were investigated to ascertain the clinical usefulness of SBCE.

## Materials and methods

### Clinical and SBCE data accumulation

The present study was based on a collection of nationwide SBCE data from institutions specializing in inflammatory bowel diseases (IBD). The inclusion criteria were (1) a negative history of recent NSAIDs use (within 2 months), (2) a suspicion of having IBD, and (3) positive small bowel mucosal injuries under total enteroscopy by SBCE at the initial diagnosis. We retrospectively accumulated clinical and SBCE data of 116 patients either with or without a final diagnosis of CD. However, eight patients were excluded after reviewing SBCE data (scarred small bowel ulcers alone in four, failure of total enteroscopy in two, and absence of small bowel mucosal lesions in two). Consequently, a total of 108 patients were the subjects of the present study.

The clinical data of each patient were obtained using a query sheet containing five queries: (1) age at the time of SBCE and sex, (2) final diagnosis, (3) indication for SBCE, (4) lesions in the gastrointestinal tract as determined by other imaging modalities at the initial diagnosis, and (5) laboratory data at the time of SBCE including the white blood cell count, hemoglobin level, platelet count, serum total protein, albumin level, and C-reactive protein level. The final diagnosis was subsequently determined based on clinical symptoms, laboratory data, and findings observed by conventional imaging modalities at each participating institution. The diagnosis of CD was based on the Japanese diagnostic criteria for CD (Supplementary Table S1) [19].

All the query sheet and full set of SBCE images were sent to our institution (Department of Medicine and Clinical Sciences, Kyushu University) and stored until analysis. The study protocol was approved by the ethics committee at Kyushu University Hospital (approved No. 24-135) and other participating institutions, and the study was conducted in accordance with the Helsinki Declaration.

### SBCE assessment

All digital video image streams of SBCE (PillCam SB2 and SB3; Given Imaging, Ltd., Yokneam, Israel) were downloaded with anonymization to the Given Imaging Reporting and Processing of Images and Data (RAPID) system. An expert capsule endoscopist (M.E.) who was blinded to the clinical information of each patient reviewed the SBCE images under the following reading protocol. SBCE images were carefully analyzed at a maximum rate of 14 frames per s with concurrent manual viewing for close assessment under the dual mode of the Multiview system.

**Fig. 1:**
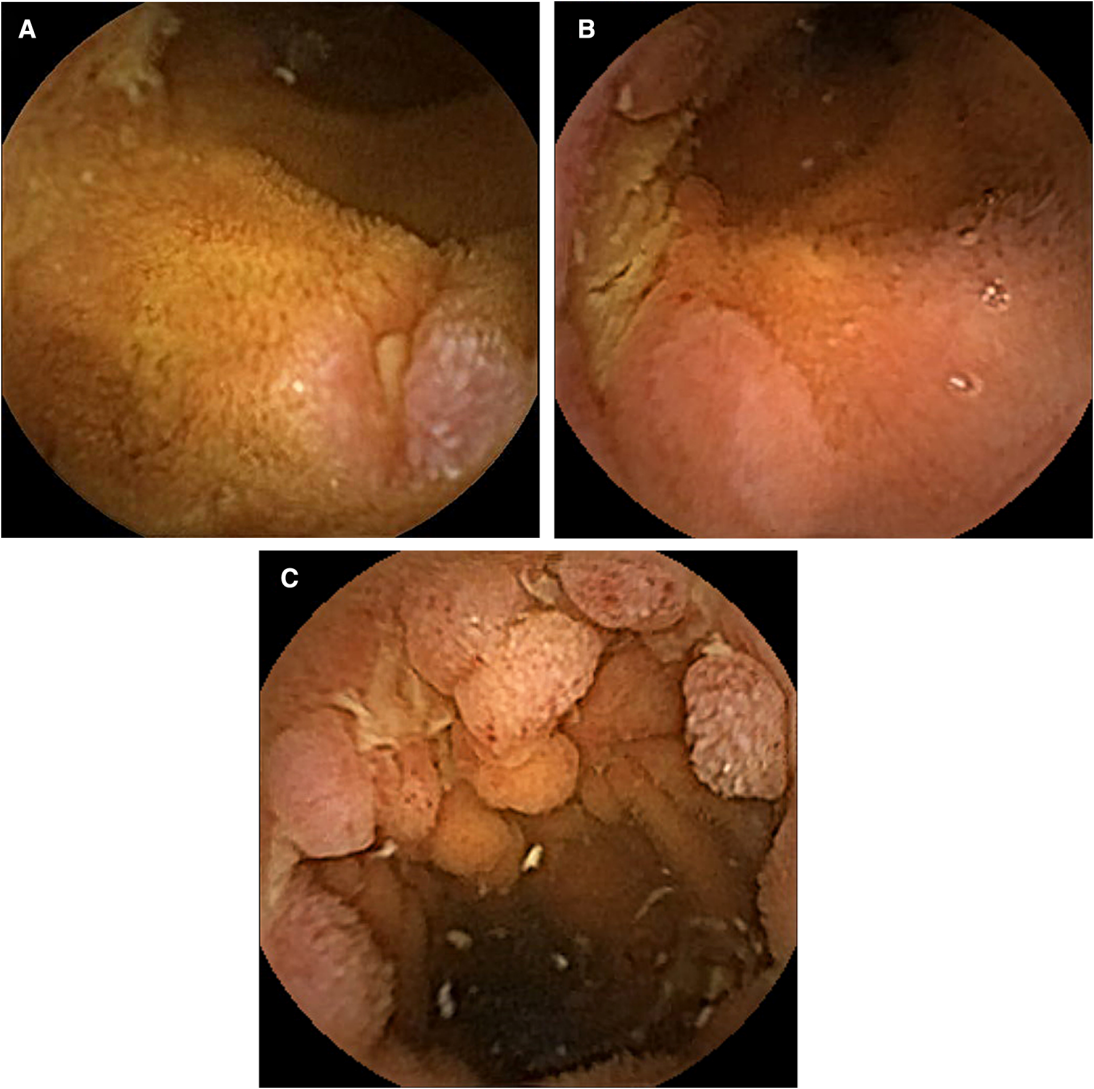
Classification of mucosal lesions under SBCE. SBCE demonstrates an **a** erosion, **b** ulcer, and **c** cobblestone appearance of the small bowel

**Fig. 2:**
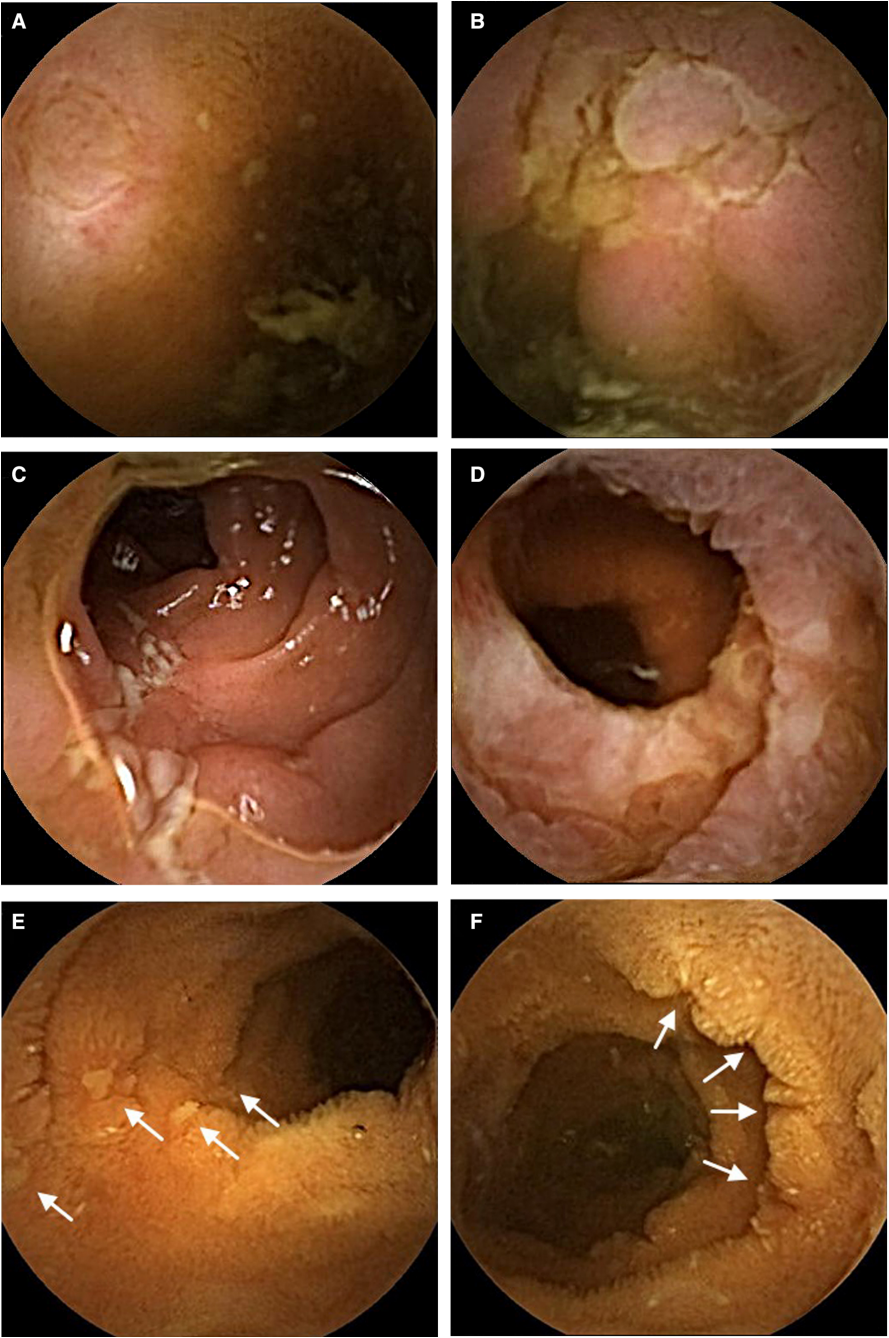
Classification of types of mucosal lesions and lesion alignment. SBCE demonstrates an **a** oval, **b** irregular, **c** longitudinal, and **d** circular ulcer. SBCE depicts, **e** longitudinal or **f** circumferential alignment of diminutive lesions (arrows)

SBCE video images were divided into three tertiles according to the small bowel transit time. Small bowel mucosal injuries were classified as erosions, ulcers, and cobblestone appearance (Fig. 1a–c). The types of erosions or ulcers were further classified as oval, irregular, linear/longitudinal, or circular (Fig. 2a–d). An erosion was defined as mucosal break of < 3 mm under SBCE, while a larger mucosal defect was defined as an ulcer [23, 24]. As for linear/longitudinal or circular mucosal injuries, the classification between erosion and ulcer was based on the minor axis (< 3 mm) of the mucosal defects. Among small bowel mucosal injuries, erosions and small ulcers of any types were defined as diminutive lesions. When multiple erosions or ulcers were observed in a patient, we determined the types of each mucosal lesion. The alignment of three or more diminutive lesions was classified as longitudinal or circumferential (Fig. 2e, f).

Finally, the overall score for small bowel mucosal injuries was calculated in accordance with the formula for the Lewis score [25]. In each tertile, small bowel cleanliness was assessed and graded on a 4-point scale according to the definition established by Brotz et al. [26].

### Assessment of inter- and intra-observer agreement

Among the 108 patients with SBCE data, we selected 25 patients with CD and 25 patients without CD for inter- and intra-observer variation analyses. The severity of small bowel mucosal injuries and the small bowel cleanliness was generally matched in each pair of SBCE. Three observers (E.M., T.M., and K.S.), who had different levels of clinical and SBCE experience, independently reviewed the SBCE data for inter-observer variation analyses. One observer (K.S.) had 2 years of clinical experience as a gastroenterologist and had read ten SBCE videos, another (T.M.) had 8 years of clinical experience as a gastroenterologist and had read 30 SBCE videos, and the remaining observer (E.M.) had 8 years of clinical experience and had read more than 200 SBCE videos. All observers were asked to review all 50 SBCE videos within 3 months following the specific reading protocol. The established protocol specified the order of SBCE videos, the maximum frame rate (14 frames per s) with concurrent manual viewing, and the modality of reading videos (dual mode). Each observer was instructed regarding the reporting protocol using representative images before viewing SBCE data, and the SBCE findings were recorded in predefined formats. For the intra-observer variation analysis, one observer (M.E.) repeatedly evaluated the identical SBCE records at an interval of more than 1 year under the same reading protocol.

All four observers were completely blinded to the patients’ final diagnosis. The results of the first SBCE analysis by an expert were used as the reference standard for the inter- and intra-observer variation analyses.

### Statistical analysis

Categorical data are expressed as frequencies and percentages, and numerical variables are expressed as mean ± standard deviation or median [interquartile range]. Clinical characteristics and SBCE findings were compared between patients with and without CD using the Chi squared test, Fisher’s exact probability test, or the Mann–Whitney *U* test, where appropriate. The Wilcoxon signed-rank test or McNemar test was used to compare SBCE findings according to the small bowel segment in patients with CD. Spearman’s rank sum test was used to calculate the correlations between the Lewis score and hematological parameters. The kappa (*κ*) statistic [27] was used to measure the agreement between each observer and the reference standard. The *κ* includes a correction for the amount of agreement that would be expected by chance alone. The *κ* index ranges from 0 (absence of agreement) to 1 (perfect agreement). A value of < 0.20 indicates slight agreement, from 0.21 to 0.40 fair agreement, from 0.41 to 0.60 moderate agreement, from 0.61 to 0.80 good agreement, and > 0.81 excellent agreement [28]. The results are reported as the mean *κ* with 95% confidence interval. The analyses were performed using JMP Pro statistical package 12.2.0 (SAS Institute, Cary, NC, USA). A *p* value of < 0.05 was considered statistically significant, and Bonferroni correction was used in the multiple comparison procedure.

## Results

### Patients

Supplementary Table S2 shows the diagnoses of the 108 patients. In addition to SBCE, ileocolonoscopy was performed in 79, esophagogastroduodenoscopy in 48, abdominal CT in 27, small bowel radiography in 21, retrograde balloon-assisted enteroscopy in 10, and abdominal US in five patients for the diagnoses. The study cohort comprised 63 patients with CD and 45 patients without CD. Final diagnoses were established in 32 patients without CD, while the diagnosis remained obscure or was IBD-unclassified in the remaining 13 patients. Patients with CD were younger and more frequently had perianal lesions than patients without CD. The serum total protein level was lower in patients without CD, whereas the serum albumin level was not different between the two groups (Supplementary Table S3).

### Comparison of SBCE findings between patients with and without CD

Table 1 compares the SBCE findings between patients with and without CD. With respect to major lesions including ulcers and cobblestone appearance, the number of ulcers was greater in patients with than without CD (9 [2–20] vs. 2 [0–7], *p* = 0.0001), and irregular ulcers (84% vs. 60%, *p* = 0.007) and longitudinal ulcers (78% vs. 20%, *p* < 0.0001) were more frequent in patients with than without CD. Cobblestone appearance was found more frequently in patients with CD (33% vs. 2%, *p* < 0.0001). The Lewis score was higher in patients with than without CD (450 [225–1012] vs. 225 [68–525], *p* = 0.0055). The number of erosions was greater in patients with than without CD (42 [18–93] vs. 8 [4–25], *p* < 0.0001), and irregular (89% vs. 64%, *p* = 0.0037) and linear erosions (90% vs. 38%, *p* < 0.0001) were more frequent in patients with CD. With respect to the alignment of diminutive lesions, both longitudinal alignment (56% vs. 7%, *p* < 0.0001) and circumferential alignment (75% vs. 9%, *p* < 0.0001) were more frequent in patients with than without CD.

**Table 1:** Comparison of SBCE findings between patients with and without CD

	CD group	Non-CD group	*p* value
Number of ulcer	9 [2–20]	2 [0–7]	0.0001
Types of ulcer			
Oval	18 (29%)	14 (31%)	0.83
Irregular	53 (84%)	27 (60%)	0.0071
Longitudinal	49 (78%)	9 (20%)	< 0.0001
Circular	12 (19%)	8 (18%)	1.0
Cobblestone appearance	21 (33%)	1 (2%)	< 0.0001
Number of erosion	42 [18–93]	8 [4–24.5]	< 0.0001
Types of erosion			
Oval	47 (75%)	32 (71%)	0.83
Irregular	56 (89%)	29 (64%)	0.0037
Linear	57 (90%)	17 (38%)	< 0.0001
Circular	21 (33%)	9 (20%)	0.19
Alignment of diminutive lesions			
Longitudinal	35 (56%)	3 (7%)	< 0.0001
Circumferential	47 (75%)	4 (9%)	< 0.0001
Lewis score	450 [225–1012]	225 [68–525]	0.0055
Bowel cleanliness score	9 [8–10]	9 [7–10.5]	0.77

The sum of the bowel cleanliness score of the three tertiles was not different between the two groups (9 [8–10] vs. 9 [7–10.5], *p* = 0.77).

### Comparison of SBCE findings among small bowel segments in patients with CD

Table 2 compares the SBCE findings among the three tertiles of the small bowel in patients with CD (Table 2). The positive rate of ulcers was significantly higher in the 3rd tertile than in the 1st and the 2nd tertiles (*p* < 0.0001 and *p* = 0.0001, respectively). When the rate of each type of ulcer was compared, irregular ulcers (1st tertile vs. 3rd tertile, *p* = 0.0001 and 2nd tertile vs. 3rd tertile, *p* = 0.0011) and longitudinal ulcers (1st tertile vs. 3rd tertile, *p* < 0.0001 and 2nd tertile vs. 3rd tertile, *p* = 0.0001) were more frequent in the 3rd tertile of the small bowel. However, the rate of cobblestone appearance was not different among the three tertiles. The rate of erosions was high in all three tertiles of the small bowel, regardless of the type. When compared the positive rate of regular alignment of diminutive lesions, circumferential (1st tertile vs. 3rd tertile, *p* = 0.0005, and 2nd tertile vs. 3rd tertile, *p* = 0.0043) and longitudinal (1st tertile vs. 3rd tertile, *p* = 0.0017, and 2nd tertile vs. 3rd tertile, *p* = 0.049) alignment were more frequent in the 1st tertile of the small bowel.

**Table 2:** Comparison of SBCE findings among three tertiles of the small bowel in patients with CD

	1st tertile	2nd tertile	3rd tertile
Presence of any ulcers	29 (46%)	32 (51%)	51 (81%)
Types of ulcer			
Oval	6 (10%)	7 (11%)	15 (24%)
Irregular	24 (38%)	30 (48%)	46 (73%)
Longitudinal	16 (25%)	25 (40%)	44 (70%)
Circular	3 (5%)	5 (8%)	7 (11%)
Cobblestone appearance	8 (13%)	12 (19%)	11 (17%)
Presence of any erosions	52 (83%)	58 (92%)	61 (97%)
Types of erosion			
Oval	29 (46%)	35 (56%)	32 (51%)
Irregular	36 (57%)	38 (60%)	47 (75%)
Linear	38 (60%)	39 (62%)	46 (73%)
Circular	7 (11%)	10 (16%)	10 (16%)
Alignment of diminutive lesions			
Longitudinal	25 (40%)	18 (29%)	9 (14%)
Circumferential	32 (51%)	24 (38%)	10 (16%)
Bowel cleanliness score	4 [3, 4]	3 [3, 4]	2 [2, 3]

In terms of bowel cleansing, the bowel cleanliness score was significantly higher in the 1st tertile than in the 2nd and 3rd tertiles (4 [3, 4] in the 1st tertile, 3 [3, 4] in the 2nd tertile, and 2 [2, 3] in the 3rd tertile, respectively).

### Correlation between laboratory data and SBCE findings in patients with CD

As shown in Table 3, there was a moderate inverse correlation between the serum albumin level and the Lewis score in patients with CD. This correlation became more significant in patients with CD of isolated small bowel disease. However, no significant correlation was found between the other laboratory data and the Lewis score.

**Table 3:** Correlation of hematological parameters and the Lewis score in patients with CD

Hematological parameter	Correlation coefficient (*ρ*)	
	All (*n* = 63)	Ileitis only (*n* = 24)
White blood cell	− 0.0174	− 0.0030
Hemoglobin	− 0.3274	− 0.3763
Platelet	0.30110	0.2628
Total protein	− 0.0340	− 0.1212
Albumin	− 0.4814	− 0.5343
C-reactive protein	0.3566	0.2428

### Inter- and intra-observer variations

The final diagnosis and the comparison of clinical characteristics of the selected 25 pairs of patients with SBCE data are shown in Supplementary Tables S4 and S5. The Lewis score and sum of bowel cleanliness score were not different between the patients with and without CD selected for the validation study.

The inter-observer agreement between the observers and the reference standard is summarized in Table 4. The *κ* value for irregular ulcers ranged from 0.35 to 0.62, showing good agreement between the two observers with greater clinical experience. However, the *κ* value for longitudinal ulcers ranged widely from 0.05 to 0.59, resulting in moderate agreement in the observer with greater clinical and SBCE reading experience. In contrast, the *κ* value for cobblestone appearance was similar, showing moderate agreement among all three observers. With respect to the alignment of diminutive lesions, the *κ* values for longitudinal and circumferential alignments improved with greater clinical and SBCE reading experience. However, the *κ* value for diminutive lesions showed poor agreement in all observers.

**Table 4:** Inter- and intra-observer agreement between the observers and reference standard

SBCE findings	Inter-observer agreement^a^			Intra-observer agreement^a^
	Observer 1	Observer 2	Observer 3	Expert endoscopist
Ulcer				
Oval	− 0.004 (− 0.22–0.22)	0.51 (0.26–0.77)	0.05 (− 0.23–0.33)	0.32 (0.04–0.59)
Irregular	0.35 (0.15–0.55)	0.64 (0.42–0.87)	0.62 (0.40–0.84)	0.53 (0.27–0.78)
Longitudinal	0.05 (− 0.04–0.14)	0.34 (0.09–0.59)	0.59 (0.37–0.80)	0.76 (0.58–0.94)
Circular	0.31 (0.03–0.58)	0.43 (0.14–0.72)	0.54 (0.26–0.82)	0.63 (0.38–0.88)
Cobblestone appearance	0.50 (0.16–0.85)	0.50 (0.16–0.85)	0.47 (0.21–0.73)	0.83 (0.61–1)
Erosion				
Oval	0.07 (− 0.12–0.26)	0.19 (− 0.11–0.49)	− 0.12 (− 0.34–0.10)	0.42 (0.10–0.73)
Irregular	0.30 (− 0.01–0.61)	0.25 (− 0.05–0.55)	0.38 (0.09–0.66)	0.49 (0.21–0.78)
Linear	0.07 (− 0.09–0.22)	0.17 (− 0.03–0.38)	0.38 (0.10–0.66)	0.57 (0.35–0.80)
Circular	0.25 (− 0.02–0.53)	0.29 (0.03–0.56)	0.44 (0.17–0.72)	0.49 (0.21–0.75)
Alignment of diminutive lesions				
Longitudinal	0.14 (− 0.04–0.31)	0.44 (0.19–0.69)	0.57 (0.36–0.78)	0.74 (0.54–0.93)
Circumferential	0.30 (0.08–0.53)	0.32 (0.07–0.58)	0.45 (0.21–0.69)	0.72 (0.53–0.91)

^a^Values are expressed as *κ* value (95% CI)

The results of intra-observer agreement for the expert capsule endoscopist are shown in Table 4. The *κ* values for longitudinal ulcers, circular ulcers, and cobblestone appearance were 0.76, 0.63, and 0.83, respectively, indicating good or excellent agreement. The *κ* values for longitudinal and circumferential alignment of diminutive lesions were 0.72 and 0.74, respectively, showing good agreement. However, the *κ* value for erosions showed only moderate agreement.

### Diagnostic accuracy of SBCE findings for CD

The diagnostic accuracy of SBCE findings for CD according to the reference standard is summarized in Table 5. The specificity and positive predictive value (PPV) for longitudinal ulcers or cobblestone appearance were favorable. However, these values for regular alignment of diminutive lesions were much higher. While the sensitivity and negative predictive value (NPV) for linear erosions were favorable, the specificity and PPV remained low. When linear erosions and regular alignment of diminutive lesions were combined, no additive effect was found on the accuracy for the diagnosis of CD.

**Table 5:** Diagnostic accuracy of SBCE findings for CD

SBCE findings	Sensitivity (%)	Specificity (%)	PPV (%)	NPV (%)
Longitudinal ulcer or cobblestone appearance	77	80	84	72
Linear erosion	90	62	77	82
Lesion alignment only				
Longitudinal	56	93	92	60
Circumferential	75	91	92	72
Longitudinal or circumferential	79	87	89	75
Linear erosion plus lesion alignment				
Longitudinal	54	91	89	59
Circumferential	71	93	94	70
Longitudinal or circumferential	75	87	89	71

*PPV* Positive predictive value, *NPV* negative predictive value

## Discussion

With the increasing use of SBCE in daily clinical practice, this procedure has been suggested to be useful in the diagnosis and management of a variety of disorders such as obscure gastrointestinal bleeding, polyposis syndromes, and IBD [1–4]. CD is considered an appropriate indication for SBCE, and higher diagnostic yields of SBCE in patients with suspected or established CD have been demonstrated when compared with other imaging modalities including small bowel radiography, CT enterography, and push enteroscopy [11–16]. However, as noted by Doherty et al. [29], the significantly higher yields of SBCE than of other imaging modalities may be partly attributable to false positives because of the lack of a precise definition of CD under SBCE. Actually, the presence of > 3 ulcers under SBCE as the threshold for suspected CD reportedly yields a PPV of up to 50% for the diagnosis of CD [30]. We, therefore, investigated endoscopic findings that may lead to better diagnostic accuracy of CD under SBCE.

In the present study, a recent history of NSAIDs use was set to be one of the exclusion criteria following the previous studies. Actually, the most widely used but not validated criteria of SBCE for the diagnosis of CD by Mow et al. [17] are defined as > 3 ulcerations under SBCE and a negative history of NSAIDs use. Such an exclusion criterion might cause selection bias; however, it seems reasonable to consider the fact that NSAIDs use is usually checked at medical interview when diagnosing CD in daily clinical practice.

We initially compared SBCE findings between patients with and without CD to clarify the characteristic findings of CD. We found that major lesions such as irregular ulcers, longitudinal ulcers, and cobblestone appearance were more frequent in patients with CD. This result seems to be reasonable because the latter two findings are defined as the major items of the Japanese diagnostic criteria for CD (Supplementary Table S1) [19]. The present study also indicated that linear or irregular erosions and longitudinal or circumferential alignment of diminutive lesions were more frequent in patients with than without CD. Considering that linear erosions and circumferential alignment of mucosal lesions are not specified in the Japanese diagnostic criteria for CD [19], such findings appear to be noteworthy for the diagnosis of CD under SBCE.

We subsequently investigated the inter-observer and intra-observer agreement of SBCE findings to determine their validity. In the inter-observer variation analysis, three observers with different levels of clinical and SBCE reading experience were selected to ascertain the influence of clinical skills on the detection of SBCE findings. We found that longitudinal ulcers and cobblestone appearance achieved moderate agreement but that irregular or linear erosions had poor agreement. The inter-observer agreement for circumferential or longitudinal alignment improved as clinical experience increased. Combined with the results of the intra-observer variation analyses, regular alignment of diminutive lesions seems to be the most useful finding for the diagnosis of CD under SBCE. Poor inter-observer agreement for each type of erosion may be partly explained by the difference in the interpretation of diminutive lesions among the observers, because the configuration of those lesions can be easily influenced by peristalsis or the amount of intestinal fluid under SBCE.

In addition to the longitudinal alignment, circumferential alignment of the diminutive lesions, especially in the 1st tertile of the small bowel, was more frequent in CD, which has not been considered as characteristic by balloon-assisted enteroscopy. Such an inconsistent result might be caused by the following reasons; first, antegrade double-balloon enteroscopy is rarely performed in CD considering the predominant involvement of the ileum. Second, SBCE images observed under physiological condition could facilitate circumferentially aligned diminutive lesions. However, multiple notching on the duodenal mucosal folds is considered as one of the characteristic findings of the upper GI tract in CD [31], which considerably mimics this type of regular alignment. Therefore, circumferential alignment as well as longitudinal one of the diminutive lesions is considered as the characteristic findings of the upper small bowel including the duodenum in CD.

We also investigated the diagnostic accuracy of CD based on SBCE findings and found higher specificity and PPVs for circumferential or longitudinal alignment than for longitudinal ulcers or cobblestone appearance. Such superiority of circumferential or longitudinal alignment might also be attributed to the visibility in the mucosal lesions, because circumferential or longitudinal alignment was mainly found in the 1st tertile of the small bowel, where the bowel cleansing level was generally good. Conversely, longitudinal ulcers were mainly found in the 3rd tertile of the small bowel, where the presence of intestinal turbid fluid or residues frequently hampers the detection of mucosal lesions under SBCE. From this viewpoint, it seems reasonable to focus on the regular alignment of diminutive lesions in the 1st tertile of the small bowel under SBCE for the diagnosis of CD.

When we compared mucosal lesions among the three segments of the small bowel in patients with CD, nearly half of the patients had major lesions in the upper two-thirds of the small bowel. This result is consistent with previous reports showing a high incidence of jejunal involvement under SBCE [32, 33]. The present study also demonstrated a considerably high incidence of diminutive lesions throughout the small bowel. Because jejunal involvement has been suggested to be a possible poor prognostic factor for small bowel CD [33], additional studies investigating the clinical impact of jejunal involvement according to the type of lesions seem necessary.

We also analyzed the possible association between hematological parameters and the Lewis score in patients with CD, for whom only a moderate inverse correlation between the serum albumin level and the Lewis score was identified. He et al. [34] recently investigated the relationship of the Lewis score with clinical disease activity indices and the C-reactive protein level in pediatric and adult patients with CD showed a weak and moderate correlation with the Lewis score, respectively. In contrast, Kopylov et al. [35] performed a prospective study of patients with quiescent or mildly symptomatic small bowel CD and reported that only a minority of patients with CD exhibiting clinical and biomarker remission (4.7%) achieved small bowel mucosal healing. It thus can be assumed that small bowel endoscopic evaluation is crucial for the precise evaluation of small bowel disease activity in patients with CD, while further studies comparing small bowel endoscopic activity with fecal biomarkers such as calprotectin or lactoferrin are required [36–38].

The present study has some limitations. First, it was conducted in a retrospective fashion. However, the observers analyzed anonymized SBCE data and were blinded to the clinical information. We thus believe that the retrospective nature of the study had a minimal effect on our results. Second, the study subjects were limited to the patients having positive small bowel mucosal injuries under SBCE, while the major advantage of the procedure is to exert high NPV for the diagnosis of small bowel CD [1–3]. However, the present study aimed to identify SBCE findings that could facilitate the identification of early CD. Since no such investigation has been reported so far, we believe that the present study has an impact when considering the role of SBCE for the diagnosis of CD. Third, the final diagnoses of the enrolled patients were determined by the gastroenterologists at each institution. However, we accumulated SBCE data from institutions specializing in IBD, and the diagnosis of CD is generally made according to the Japanese diagnostic criteria for CD [19]. Therefore, the diagnosis of CD in our patients is considered to have been reliable. Fourth, the patients without CD included 13 patients with an unconfirmed diagnosis. It is, therefore, possible that patients with CD who did not meet the diagnostic criteria (e.g., those with a very early stage of CD) might have been included in the group of patients without CD. However, such a misclassification of the very early stage of CD would have rather strengthened our results. Finally, we could not compare SBCE findings with other imaging modalities including balloon-assisted enteroscopy, which might hamper the reliability of our results. Prospective comparative studies are necessary, while the determination of characteristic SBCE findings for the diagnosis of CD seems also important.

In conclusion, circumferential or longitudinal alignment of diminutive lesions, especially in the upper small bowel, under SBCE can be a clue for the diagnosis of CD, while a certain level of clinical and SBCE experience is mandatory for accurate detection. Further prospective studies are necessary to ascertain the clinical usefulness of SBCE and determine its true diagnostic yield in patients with suspected CD.

## Electronic supplementary material

Below is the link to the electronic supplementary material.

Supplementary material 1 (DOCX 27 kb)

## Abbreviations

SBCE: Small bowel capsule endoscopy
CD: Crohn’s disease
IBD: Inflammatory bowel diseases
NSAIDs: Nonsteroidal anti-inflammatory drug
