# Supplementary material for: Capsule endoscopy findings for the diagnosis of Crohn’s disease: a nationwide case–control study
Source: J Gastroenterol. 2018 Sep 15;54(3):249–60. doi: 10.1007/s00535-018-1507-6 (PMC6394710; doi:10.1007/s00535-018-1507-6)
Supplement: Supplementary file 1 — Supplementary material 1 (DOCX 27 kb) [file 535_2018_1507_MOESM1_ESM.docx]

Supplementary Table S1. Proposed Japanese diagnostic criteria for CD^19)^

|  | 1) Major findings |  |
| --- | --- | --- |
|  | 1. Longitudinal ulcer^a^ |  |
|  | 1. Cobblestone-like appearance |  |
|  | 1. Noncaseating epithelioid cell granuloma^b^ |  |
|  | 2) Minor findings |  |
|  | 1. Irregular-shaped and/or quasi-circular ulcers or aphthous ulcerations found extensively in the gastrointestinal tract^c^ |  |
|  | 1. Characteristic perianal lesions^d^ |  |
|  | 1. Characteristic gastric and/or duodenal lesions^e^ |  |
|  |  |  |
|  | Definite |  |
|  | 1. Major finding A or B^f^ |  |
|  | 1. Major finding C, with minor finding a or b |  |
|  | 1. All minor findings a, b, and c |  |
|  | Suspected |  |
|  | 1. Major finding C, with minor finding c |  |
|  | 1. Major finding A or B, but cannot be differentiated from ischemic colitis or ulcerative colitis |  |
|  | 1. Major finding C only^g^ |  |
|  | 1. One or two minor findings |  |

^a^ In the small bowel, the ulcer occurs more commonly on the mesenteric side.

^b^ The rate of detection improves by creating serial sections.

^c^ In typical cases, the ulcers are arranged longitudinally, but this does not occur in some cases. It is necessary that they persist for at least 3 months. It is necessary to exclude enteric tuberculosis, entero-Behçet’s disease, simple ulcers, nonsteroidal anti-inflammatory drug (NSAID)-induced ulcers, and infectious enterocolitis.

^d^ These lesions comprise anal fissures, cavitating ulcers, anal fistulas, perianal abscesses, and edema-like and skin tags.

^e^ These lesions have a bamboo joint-like appearance with notch-like depression.

^f^ In cases with only longitudinal ulcers, it is necessary to exclude ischemic intestinal lesions and ulcerative colitis. In cases with only a cobblestone-like appearance, it is necessary to exclude ischemic intestinal lesions.

^g^ It is necessary to exclude inflammatory diseases with granulomas such as intestinal tuberculosis.

Supplementary Table S2. Clinical diagnoses of 108 patients

|  | CD group | 63 |  |  |
| --- | --- | --- | --- | --- |
|  | Ileitis type |  | 24 |  |
|  | Ileocolitis type |  | 39 |  |
|  | Non-CD group | 45 |  |  |
|  | Entero-Behçet disease |  | 9 |  |
|  | Ulcerative colitis |  | 5 |  |
|  | Intestinal tuberculosis |  | 3 |  |
|  | Aphthoid enteritis |  | 3 |  |
|  | Eosinophilic gastroenteritis |  | 2 |  |
|  | Amebic enteritis |  | 1 |  |
|  | Cryoglobulinemic vasculitis |  | 1 |  |
|  | Cytomegalovirus enteritis |  | 1 |  |
|  | Giardiasis |  | 1 |  |
|  | Simple ulcer |  | 1 |  |
|  | Gastrinoma |  | 1 |  |
|  | Malignant lymphoma |  | 1 |  |
|  | Acute enteritis |  | 1 |  |
|  | Inflammatory bowel disease unclassified |  | 1 |  |
|  | Protein losing enteropathy |  | 1 |  |
|  | Irritable bowel syndrome |  | 1 |  |
|  | Unconfirmed |  | 12 |  |

Supplementary Table S3. Comparison of clinical characteristics between patients with and without CD

|  |  | CD group | Non-CD group | P value |  |
| --- | --- | --- | --- | --- | --- |
|  | Mean age [±SD] | 25.8±11.5 | 43.4±19.0 | <0.0001 |  |
|  | Male: female | 44:19 | 31:14 | 1.0 |  |
|  | Clinical symptoms suspected of CD | |  |  |  |
|  | Abdominal symptoms | 50 (79%) | 34 (76%) | 0.47 |  |
|  | Perianal lesion | 24 (38%) | 6 (13%) | <0.005 |  |
|  | Extraintestinal complication | 18 (29%) | 15 (33%) | 0.68 |  |
|  | Hematological data* |  |  |  |  |
|  | White blood cell (/μl) | 7430 | 7880 | 0.60 |  |
|  | Hemoglobin (g/dl) | 12.6 | 12.4 | 0.59 |  |
|  | Platelet (x10^4^/μl) | 34.7 | 31.8 | 0.18 |  |
|  | Total protein (g/dl) | 7.2 | 6.7 | <0.01 |  |
|  | Albumin (g/dl) | 3.7 | 3.6 | 0.77 |  |
|  | C-reactive protein (mg/l) | 22 | 19 | 0.09 |  |

*Each hematological parameter is expressed as the mean value.

Supplementary Table S4. Clinical diagnoses of 25 pairs of patients

|  | CD group | 25 |  |  |
| --- | --- | --- | --- | --- |
|  | Ileitis type |  | 12 |  |
|  | Ileocolitis type |  | 13 |  |
|  | Non-CD group | 25 |  |  |
|  | Entero-Behçet disease |  | 8 |  |
|  | Ulcerative colitis |  | 5 |  |
|  | Intestinal tuberculosis |  | 3 |  |
|  | Eosinophilic gastroenteritis |  | 2 |  |
|  | Amebic enteritis |  | 1 |  |
|  | Cryoglobulinemic vasculitis |  | 1 |  |
|  | Giardiasis |  | 1 |  |
|  | Simple ulcer |  | 1 |  |
|  | Gastrinoma |  | 1 |  |
|  | Malignant lymphoma |  | 1 |  |
|  | Acute enteritis |  | 1 |  |

Supplementary Table S5. Comparison of clinical characteristics between patients with and without CD for the validation study

|  |  | CD group | Non-CD group | P value |  |
| --- | --- | --- | --- | --- | --- |
|  | Mean age [±SD] | 22.6±8.4 | 50.3±18.8 | <0.0001 |  |
|  | Male: female | 16:9 | 18:7 | 0.76 |  |
|  | Clinical symptoms suspected of CD | |  |  |  |
|  | Abdominal symptoms | 22 (88%) | 19 (76%) | 0.46 |  |
|  | Perianal lesion | 11 (44%) | 0 (0%) | <0.001 |  |
|  | Extraintestinal complication | 8 (32%) | 10 (40%) | 0.77 |  |
|  | Hematological data* |  |  |  |  |
|  | White blood cell (/μl) | 7705 | 8770 | 0.44 |  |
|  | Hemoglobin (g/dl) | 12.6 | 12.1 | 0.33 |  |
|  | Platelet (x10^4^/μl) | 36.5 | 30.4 | 0.14 |  |
|  | Total protein (g/dl) | 7.1 | 6.4 | 0.017 |  |
|  | Albumin (g/dl) | 3.6 | 3.2 | 0.12 |  |
|  | C-reactive protein (mg/l) | 20 | 25 | 0.93 |  |

*Each hematological parameter is expressed as the mean value.
